# Recovery from shift work

**DOI:** 10.3389/fneur.2023.1270043

**Published:** 2023-11-02

**Authors:** Alexandros Kalkanis, Saartje Demolder, Dimitrios Papadopoulos, Dries Testelmans, Bertien Buyse

**Affiliations:** ^1^Department of Respiratory Diseases, Louvain University Center for Sleep and Wake Disorders (LUCS), University Hospitals Leuven, KU Leuven, Leuven, Belgium; ^2^Laboratory of Respiratory Disease and Thoracic Surgery (BREATH), KU Leuven-University, Leuven, Belgium

**Keywords:** shift work, shift work disorder, circadian rhythm disorder, recovery, interventions

## Abstract

One fifth of today's workforce is engaged in shift work and exposed to various mental and physical health risks including shift work disorder. Efficiently recovering from shift work through physical and mental interventions allows us to mitigate negative effects on health, enables a better work-life balance and enhances our overall wellbeing. The aim of this review is to provide a state-of-the-art overview of the available literature. The role of sleep timing and naps, light therapy and psychotherapy, diet and exercise in recovery from shift work is presented here. We further review the impact of shift schedules and social support on post-shift unwinding.

## 1. Introduction

Shift work plays an important role in today's workforce due to the continuous demand for availability and customer service in our economy as well as its role in public safety and health while offering various financial benefits. It's an essential work format in the health, transport, industry, commerce, and hospitality sectors (1). In total 21% of workers included in the sixth European Working Conditions Survey in 2015 reported engaging in shift work (1). The percentage of European workers aged 20 to 64 years old who regularly performed shift work in 2021 ranged from 6.8 to 33.6% (2) and thus stays somewhat similar to 2015. An increase in shift work in the Benelux over the last 10 years has also been observed (2). Shift work is defined in literature as work conducted outside the standard daylight working hours (7/8 am to 5/6 pm) and can entrail fixed or rotating shifts (1, 3). Shift work may thus expose workers to light during normal sleeping hours which disrupts normal sleeping patterns and causes circadian misalignment (4, 5). The change in the interaction between circadian and homeostatic processes when working a night shift leads to sleep loss, excessive sleepiness, and impaired alertness during work (6). Not surprisingly, an increased occupational and motor accident risk, the latter especially after night shifts, has been found, which can negatively impact the health of the shift worker and others involved (7, 8). A plethora of other health issues have been described concerning shift work as well.

Shift work disorder, a specific sleep disorder related to irregular work schedules, may affect up to 10% of shift workers (9). According to the International Classification of Sleep Disorders the prevalence of shift work sleep disorder is estimated to be 10 to 38% of workforce (10, 11). This disorder encompasses a persistent circadian-related sleep problem resulting in insomnia and/or excessive sleepiness in shift workers for at least 3 months, accompanied by a reduction in total sleep time. Moreover an increased risk of poor mental health (12), as well as a higher risk for the development of metabolic syndrome (13, 14), cardiovascular disease, in particular coronary heart disease (15), and gastrointestinal disease such as peptic ulcers (16, 17) have been reported. It seems vital that we seek interventions to limit or erase the negative impact of shift work on health. In this review, we will describe the recent literature on non-pharmacological as well as pharmacological (melatonin) interventions that can aid shift workers in their recovery from work. Recovery from work has been defined as the process of psychophysiological unwinding after effort expenditure and has been described to mitigate some of the negative health risks mentioned above (18). As health professionals, it's important to minimize negative health outcomes for such a large proportion of our workforce including our colleagues.

## 2. Role of sleep and circadian rhythms

Sleep regulation is guided by the interaction between a homeostatic process, reflected in the amount of slow wave sleep, representing the sleep pressure accumulated during wakefulness, and a circadian process showing 24-h rhythmicity entrained to the light-dark cycle. Shift work induces sleep disturbances by disrupting the temporal relation between the two processes due to the adoption of irregular sleep schedules that are not aligned with the internal circadian clock and the external light-dark cycle (5). Shift workers suffer from chronic sleep deprivation, impaired sleep quality, and symptoms of insomnia or excessive daytime sleepiness that eventually could lead to alertness or cognitive deficits (19, 20). Obtaining sufficient and high-quality sleep after night shifts has been shown to enhance post-work recovery from fatigue in nurses (21, 22).

### 2.1. Traditional countermeasures

#### 2.1.1. Recovery from sleep debt

Hypnotics and melatonin have been studied as means to extend sleep duration after night shifts. Although these drugs increased daytime sleep among shift workers, no significant effect on sleepiness and alertness during the shift was found, while their long-term efficacy and tolerance is a matter of debate (23, 24). However, they can be considered on an intermittent basis to counterbalance the cumulative sleep debt in shift workers with insomnia complaints.

#### 2.1.2. Naps

Napping before or during the night shift may increase the total amount of sleep time obtained throughout the day and also improve performance and decrease fatigue during the shift (25). Moreover, napping during the shift resulted in a lower need for recovery after work, as has been shown in studies with nursing personnel (26–28). However, the phenomenon of sleep inertia and its negative effect on alertness immediately after waking from such naps could be a hindering factor, especially in professions where operational readiness is crucial (29).

#### 2.1.3. Stimulants

Caffeine has been extensively studied as a stimulant for consumption during night shifts to promote vigilance and performance (30). Caution is required to avoid consuming it too late in the shift as it may interfere with the daytime recovery of sleep. Other stimulants, such as modafinil and armodafinil, have also been tested with favorable results (31, 32). While all these measures may reduce performance deficits during shifts, some level of residual sleepiness may persist, especially close to the circadian temperature nadir that usually occurs at about 4 a.m.

### 2.2. Sleep timing and circadian adaptation

A crucial factor that contributes to sleep and daytime complaints of shift workers is the desynchrony between the sleep-wake schedule forced by the socio-economical commitments and the internal circadian rhythm. Although there is significant inter-individual variability regarding tolerance to shift work, with influences from genetic-epigenetic factors (33, 34), age, and chronotype (35), the diurnal nature of the circadian system drives us to be alert at daytime and sleepy at night.

**Figure 1:**
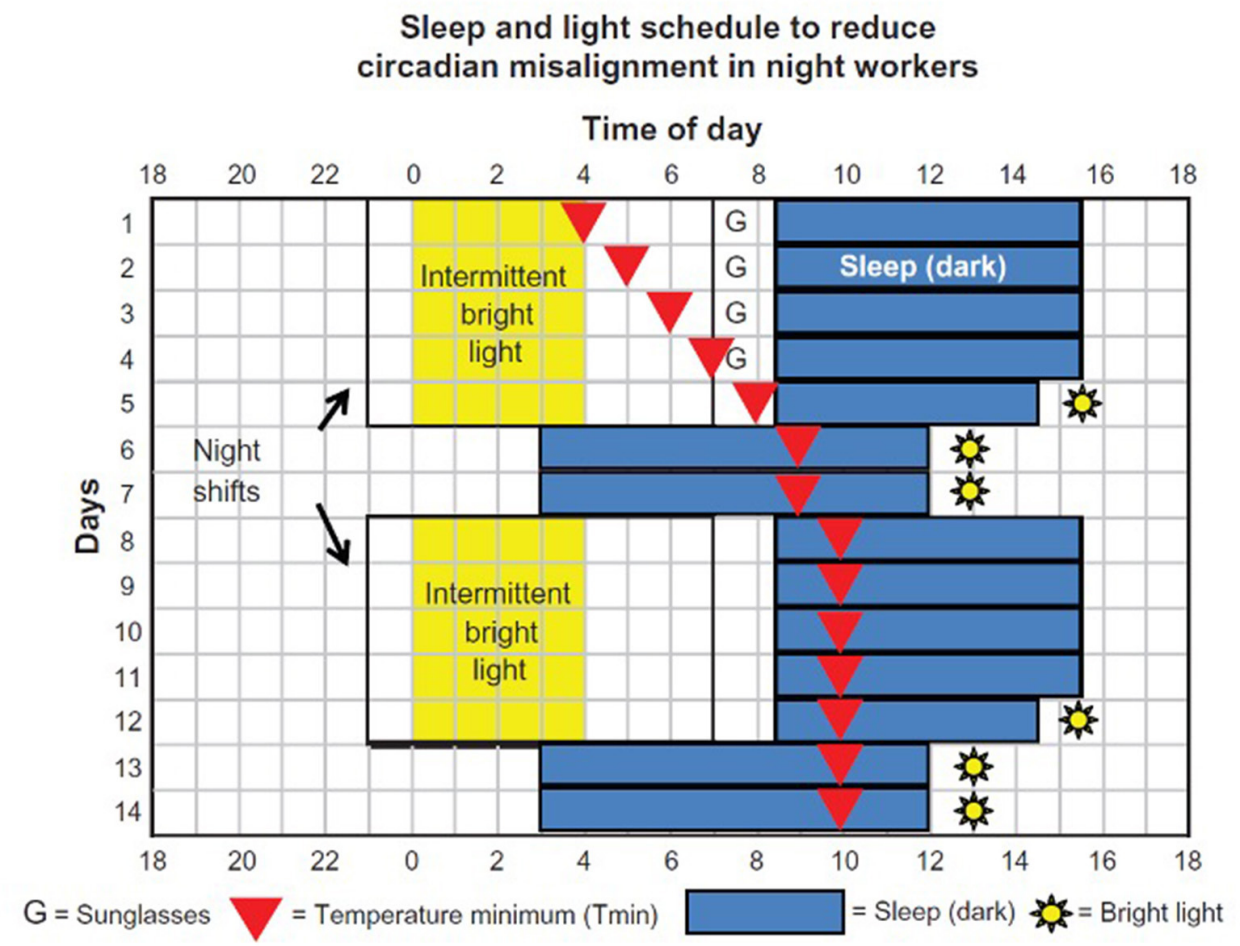
Process of phase shifting in permanent night shift workers using chronotherapy (37).

For shift workers on fixed schedules (night shifts only or rotating between evening and night shifts), chronotherapeutic approaches can be employed to phase shift the internal circadian clock and reduce the circadian misalignment (36). With this approach, the night-shift worker should aim to go to bed as early as possible after the shift to avoid longer exposure to morning light and excessive delay of the temperature nadir. A shift worker should try to obtain at least 7 h of sleep in a dark and quiet environment, except for the morning on his 1^st^ day off after consecutive night shifts, when he should sleep somewhat shorter to build up sleep pressure for the following night. On days off, the worker should adopt a later bedtime and rise time, so the shifted temperature nadir remains inside the sleep period, and aim for nearly 9 h of continuous sleep, based on individual needs (Figure 1) (37). Apart from sleep timing, phase shifting of the light-dark cycle, using timed exposure to bright light during the night shift, is additionally important to facilitate circadian adaptation (37). In the same context, melatonin can also be used for phase shifting along with its soporific properties. Melatonin advances the circadian clock when taken in the afternoon/evening (for night workers who prefer to sleep before the shift) and delays it when consumed in the morning (for those who prefer to sleep after the shift) (38).

Chronotherapy, however, is not an option for shift workers on fast-rotating schedules that include day and night shifts and constitute the majority of shift systems in modern workplaces, especially in the health sector. For these workers, a combination of general behavioral approaches (sleep hygiene practices, scheduled naps, caffeine), timed light exposure and avoidance, melatonin use, and adequate planning of days off for recovery could apply to reduce the adverse effects of the shift work-induced circadian misalignment (39).

## 3. The effect of shift schedules on recovery

Apart from individual differences in adaptability to shift work, the specific shift schedules can also have an impact on the recovery process. Recovery can be defined as “the period of time that an individual requires to return to a normal or pre-stressor level of functioning following the termination of a stressor” (40). It can be assessed either behaviorally (performance, sleep, mood, wellbeing) or biologically (autonomic activity indicators, such as blood pressure and heart rate variability). An early report from Totterdell et al. (40) highlighted that most behavioral recovery measures were at their worst on the first rest day after a night shift in nurses and gradually improved on subsequent days, highlighting the need for two recovery days following 1 or more night shifts. Similarly, Malmberg et al. (41) concluded that physicians require two nights' sleep for a full recovery from a night call. Chung et al. (42) measured heart rate variability during sleep in nurses working on a rapidly rotating 3-shift schedule and found that 2 consecutive days off were needed to restore sleep-related autonomic regulation. However, other studies have reported shorter (43) or longer (44) periods for recovery than the conventional rule of having 2 days off. The direction of the rotating shift system has also been found to play a role in the recovery process, favoring forward over backward rotating schedules (45).

Past research has also tried to identify differences in recovery between various permanent night shift systems, namely the 2 + 2, 4 + 4, and 7 + 7 (night shifts + recovery days) schedules: while behavioral recovery measures favored the 7 + 7 schedule (46), autonomic function restoration was higher in the 2 + 2 schedule (47). More studies are needed to clarify which night shift schedule is more appropriate in terms of recovery.

## 4. The effect of other sleep disorders on recovery

Recovery from shift work can be more challenging when comorbid sleep disorders, causing excessive sleepiness, such as obstructive sleep apnea (OSA) and narcolepsy, are present or when maladaptive behaviors and cognitions about sleep lead to insomnia (10). Referral to a sleep specialist is crucial to differentiate between OSA and shift work sleep disorder, especially since both patient groups can score equally high on the Epworth Sleepiness Scale (ESS) questionnaire (48). Prevalence of OSA in shift workers is estimated in a recent systematic review between 14.3 and 38.1%, while only between 1 and 14% in the general adult population (49, 50). We note that another recent meta-analysis could not find an increased OSA prevalence in shift workers, although this finding was limited as not all included studies used polysomnography to diagnose OSA (9). To fully observe this effect, polysomnography during the daytime after a night shift is more meaningful than a classical nighttime polysomnography (51). Underdiagnosing OSA by performing a study during an unusual for the patient sleep period, may lead to under treatment. The effect of OSA treatment in this group needs to be monitored because remaining sleepiness might be caused by shift work sleep disorder.

## 5. Light and need for recovery from shift work

Light is the strongest synchronizing agent (zeitgeber) for the circadian system and the most important and effective factor affecting the health of shift workers (52). Light exposure history has been shown to have an impact on light sensitivity in humans, as assessed by the magnitude of the suppression of melatonin secretion by nocturnal light (53). Shift workers and especially night workers are exposed to a substantial amount of unnatural light, which has been shown to suppress melatonin and aggravate the circadian misalignment between the internal circadian clock and activities (54).

Workers on long-lasting night shift schedules often experience a stabilized misalignment of the circadian rhythm with the day-night cycle and they appear to be less susceptible to the health risks associated with shift work (19). For these workers, attention should be paid to their nighttime alertness and preservation of their daytime recovery. According to the National Institute for Occupational Safety and Health (NIOSH) (55), increased light exposure during the first half of the shift can improve the alertness in this group. There is less concern regarding further phase delay through light exposure since their sleep period is constantly delayed. Moreover, bright light during the night shift could help preserve a stable pattern of melatonin secretion by assisting in the delay of circadian rhythms. In theory, melatonin can be secreted later to help initialize day sleep in a dark bedroom, but there is inconsistent data regarding this theory. Previous research has found that controlling light exposure can improve circadian alignment in individuals who work permanent night shifts (56). A recommended sleep-and-light schedule by Smith et al. (57) has these workers exposed to intermittent bright light during their night shifts, especially between midnight and 4:00 am, to gradually achieve through phase delay, a stable circadian rhythm. This stability is then preserved, even during the 2-day recuperation period of the weekend.

Scheduling and timing can get more complicated for employees working rotating shift schedules that cycle through progressions or who have on-call duties, including sporadic or rapidly rotating night work. In these cases, light-based intervention has been studied for both preventive and restorative purposes. A week of “preparation” with bright light during the day could alleviate the effect of light exposure during the late shift. Other studies demonstrated the same stabilizing effect after a duration of light exposure that ranged from 2 weeks to even a couple of days prior to the shift (58, 59). Studies have shown that exposure to additional light throughout the day before the shift can reduce the negative effects of evening and nocturnal light on sleep quality, melatonin levels, and circadian phase delays in healthy adults (60, 61).

Bright light can also act as a stimulant and improve alertness during evening or night shifts. A recently published systematic review and meta-analysis of 14 studies from 7 countries showed that lighting interventions, especially blue-enriched white light significantly improved the sleepiness of night-shift workers (62). A recent randomized controlled trial used the combination of evening light exposure and morning light avoidance in a group of healthy nurses who worked full-time rapidly rotating shift schedules. The researchers used the improvement of diet as a control intervention. Besides improvements in fatigue, sleepiness, and sleep duration, light interventions improved the mood and reduced the number of work errors (63).

NIOSH advises reducing light exposure during the second half of the shift, to facilitate sleep when workers get home (55). Sunglasses can block the alerting effect of daylight on the ride home and can facilitate the secretion of melatonin. On the other hand, strategies to improve health through the management of light for circadian (re) adaptation have been broadly explored. In 2019 the Working Time Society (WTS) and International Committee on Occupational Health (ICOH) published their consensus statements regarding evidence-based interventions using light to improve circadian adaptation to working hours (64). Interventions using natural or artificial light should be done extremely carefully to avoid further circadian disruption and should consider the type of shiftwork and the chronicity of time misalignment.

## 6. Diet and need for recovery after shift work

There is increasing evidence suggesting that eating time and frequency may significantly influence health of the general population, particularly in shift workers (65). Due to several factors ranging from voluntary to involuntary busy schedules, many shift workers eat at non-optimal times (66). The unpredictable nature of their work schedules can disrupt regular mealtimes, resulting in skipped or missed meals. This disruption of the circadian feeding timing can lead to erratic eating patterns and an increased tendency to opt for unhealthy food choices; many shift workers reach for convenience foods, which are often high in sugar, unhealthy fats, and lacking essential nutrients. These irregular dietary patterns of rotating shift workers were recently presented in a systematic review and meta-analysis (67).

Research indicates that shift workers tend to consume more junk food and sugary snacks compared to those on a regular 9-to-5 schedule (68). The consequences of these poor eating habits extend beyond weight gain and energy fluctuations. Irregular eating patterns have been associated with an increased risk of obesity, diabetes, cardiovascular diseases, and gastrointestinal issues (69, 70). Furthermore, inadequate nutrition can impact cognitive function, mood, and overall wellbeing. Lately, there are more data regarding the connection between nutritional circadian regulation and cancer prevention (71). Although described in the literature and widely assumed to be true, there is scarce evidence for food zeitgeber effects in human studies, as demonstrated in the recent review by Lewis et al. (72). In one study (73) the researchers succeeded in showing that meal timing has the capability of regulating the human circadian system and fulfills at least one of the zeitgeber criteria proposed by Aschoff (74).

Considering this low-grade evidence, the question remains whether adjustments to meal timing and composition could potentially reduce chronobiological strain and recovery from modern 24/7 lifestyles, such as shift work, in humans. It is accepted that timing of meals plays a vital role in optimizing energy levels and sleep quality, but when should the last meal be in relation to the first sleep period after a shift? Kogevinas et al. (75) studied the negative health outcomes of mistimed eating patterns and concluded that we should generally avoid eating 2 h or less before initiating sleep. This could be very important to the evening shift workers when they return home, to leave enough time between their last meal and the recovering sleep. Implementing this habit could be more difficult though for the night shift, because day-time sleep could be shorter and interrupted from hunger (76, 77). In a population of overweighted fixed night-shift workers, longer time interval between the last meal and sleep onset appeared to be protective against dyslipidemia (78). Unfortunately, it is an unhealthy habit of night workers to shorten the interval till the sleep recovery of the shift and this has been shown to increase the total duration of diurnal sleep (79). In a research study of nighttime nurses, every hour decrease in the interval between the last meal and sleep onset there was an increase of 0.39 h on diurnal sleep duration. This nutritional misalignment has also been connected to increased risk for obesity (80).

Besides timing, shift workers are advised to thoughtfully schedule food intake during the evening and the night and opt for a healthy snack. Eating a large meal during the nightshift could impair cognitive performance and sleepiness above the effects of time of night alone (81, 82). The feasibility of fasting during the shift remains debatable, despite the possible positive effects (83). Shift workers could benefit from a short meal or a snack for social, hedonic, and stress-related factors. Scheduling and planning of meals are more important than dramatic measures like strict fasting because shift work and especially night schedules lead to a high caloric intake, even during the recovery days.

A healthy and balanced diet is crucial for mitigating health risks and enhancing the overall quality of life for shift workers. Their diet should focus on providing sustained energy throughout their shifts. Hence, consuming nutrient-dense meals that balance carbohydrates, proteins, and healthy fats is extremely important (84). This can be challenging because studies have shown that satiety decreases after a night shift (85) leading to the described tendency to overeat and turn to junk food. The study in the group of night nurses showed that an unbalanced diet can also affect recovery as every 1 g of fat and 1 g of carbohydrate consumed in the last meal before sleep was associated with an increase in diurnal sleep onset latency of 0.13 h (79). In addition to proper nutrition, hydration is crucial for shift workers. Staying hydrated throughout the shift can help combat fatigue, maintain alertness, and support overall health (86). It is essential to prioritize water intake and limit caffeine consumption and sugary drinks, as these can disrupt sleep patterns and lead to dehydration.

Given the negative impacts of irregular shiftwork eating behaviors, correcting these habits for a healthy lifestyle is essential. Healthcare specialists have proposed different methodologies ranging from psychological to medical interventions. The self-determination theory is the foremost step in correcting such disorders (87). Setting specific goals, planning meals in advance, and incorporating healthy snacks can promote adherence to a nutritious diet (88). The Centers for Disease Control and Prevention (CDC) (55) recommends several strategies for managing work hours, including meal planning, consuming high-protein meals during the shift, and avoiding heavy meals close to bedtime. These guidelines can help shift workers optimize their nutrition, maintain energy levels, and promote better sleep quality.

## 7. Role of exercise in recovery from shift work

Shift workers face significant challenges in maintaining a healthy lifestyle due to the lack of a proper exercise routine. Factors such as irregular work schedules and consequently sleep disruptions make it difficult for individuals to find the time and motivation for physical activity (89). Shift workers often experience higher levels of acute fatigue due to irregular sleep patterns and disrupted circadian rhythms. It has been documented, for example, that nurses struggle with moderate to high fatigue during inter-shift recovery, and the situation is even worse for the nurses working 12 h shifts (90). As an individual grows older, the combination of a sedentary lifestyle and the demands of shift work can further contribute to increased fatigue, reduced cognitive function, and decreased physical performance (90). These symptoms can also be present during days-off, creating a vicious circle (43).

Physical inactivity can exacerbate fatigue among shift workers, negatively impacting their performance, productivity, and overall wellbeing. A recent systematic review and meta-analysis aimed to compare physical activity and sedentary behavior in shift workers with non-shift workers (91). Interestingly, habitual levels of physical activity were similar for shift and non-shift workers, with only 41% of shift workers meeting physical activity guidelines. The writers pointed out the heterogeneity of the included studies regarding measurement and scoring of physical activity and the population bias: 50% of the scientific work included in the review studied nurses, which might explain the relatively comparable physical activity and the low sedentary time. In reality, sedentary time is prevalent in most workplaces that involve shift work (92). Moreover, the difference between physical activity and exercise needs to be highlighted.

According to Caspersen et al. (93) exercise is a subset of physical activity that is planned, structured, and repetitive and has as a final or an intermediate objective to improve or maintain physical fitness. Due to disturbed natural circadian rhythm various physiological processes and lifestyle habits like exercise also become affected (94). The lack of regular exercise among shift workers has significant physical and mental health consequences (95). On the other hand, regular exercise could act in a preventive and recuperating way by helping shift workers establish a more stable circadian rhythm by promoting better sleep-wake patterns.

First and foremost, exercise could serve as a non-photic synchronizer of circadian rhythmicity, or a zeitgeber (96). Back et al. (97) have shown that physical exercises have non-photic effects that can positively impact the circadian timing system, thereby benefiting the health of individuals in various situations. The systematic review of Lewis et al. (98) showed that correctly timed exercise could help in maintenance of chronobiological homeostasis and improve general health in the unnatural modern work- and living environment. In this way, exercise could keep “winding” the internal clock, promoting better synchronization and improving sleep quality among shift workers. Appropriate timing of exercise may help adapt to a specific shift schedule or facilitate readaptation to a daytime schedule after the end of a shift (99).

A systematic review of physical activity-based interventions in shift workers in 2018 studied the efficacy of exercise promoting initiatives in this occupational group (100). The findings suggested that physical activity could mitigate intermediate health-risk factors in shift workers. More specific, a randomized controlled trial showed that the combination of a worksite exercise and behavioral intervention improved sleep duration and quality in shift workers (101). Regarding the timing of the intervention, it is important to mention that in this study the participant could choose from several suitable time-windows for their exercise. This is important because a lot of workers struggle to incorporate physical exercises into daily routines.

Different studies have recently tried to objectify the recovery effect of such an intervention before the shiftwork. It is generally accepted that regular exercise improves cardiovascular health and enhances physical performance, allowing shift workers to better cope with the demands of their work. High-intensity interval training prior to night shift work has shown to improve physical work capacity and endothelial and vascular function (102). These results were not reproduced though in a larger study after an 8-week intervention with supervised high intensity physical activity three times a week. Another study also failed to show that general aerobic fitness is associated with the recovery after a 24 h shift, as shown from the parasympathetic cardiac control and heart rate variability (103). There is also a concern regarding the effect of training on the sleep schedule and the recovery during the days-off, especially in a rapidly rotating shift schedule. Incorporating a dense exercise schedule into the shift rotation could further exacerbate the circadian disruptions and impair the recovery (104). While there is literature showing no disturbance of sleep quality from close-to-bedtime exercise (105, 106), a recent systematic review and meta-analysis showed that sleep could be affected after vigorous exercise ending ≤ 1 h before bedtime (107). It is generally advised that extremely intense physical exertion should be avoided before the work shift so that recovery before the upcoming shift would be optimized.

Several national health organizations suggest prioritizing recovery and rest days by exploring different exercise options: considering a workout partner, following a flexible work-out plan, incorporating shorter workouts, utilizing breaks, and even finding opportunities at work (108, 109). A recent study (110) showed that a short but continuous training of moderate intensity elicited an anti-inflammatory effect and significantly reduced sleep fragmentation in shift workers. Lack of free time due to the particularities of shift working, is a factor that keeps many employees away from frequent training. Training facilities within the workplace and exercise during the shift-breaks could be a viable alternate (111). For example, isometric and isotonic exercises during the inter-shift break positively affected fatigue recovery of the control room staff of an urban railway (111). Another possibility is a smartphone-based home workout program. Such a program for shift-work nurses implemented by Baek et al. showed statistically significant improvements in physical and psychological health (112).

Thus, while there is a lack of solid evidence about the effect of exercise on recovery, incorporating training into the daily routine of shift workers can positively impact their overall wellbeing and ability to adapt to shift work schedules.

## 8. Effect of social support on burden of shift work

Sacrificing recuperative sleep for social obligations, such as childcare or planned activities, can be challenging and is even discouraged in the literature (113), in an effort to reduce the health and safety risks of a night shift. More often, social networking and activities are sacrificed due to rotating working hours, in an effort to create free time (114). Shift work can be inherently stressful due to irregular schedules, work demands, and the disruption of personal routines. Shift work can thus often lead to social isolation due to working unconventional hours. For a shift worker, the social consequences for their partner, their family and their social circle can be even more important than the biological ones they experience themselves (115). Not every worker is affected in the same way. Certain personality traits are a better match for shift work such as flexibility, extraversion, self-esteem and hardiness (116). Everyone though, can be, to a level, impacted by the fatigue and the stress in the modern 24/7 world. Up to 88% of night shift working nurses were impacted by negative psychological effects of their work, especially females with domestic responsibilities (117). Shift work can also have severe direct implications on a worker's family life, even leading to an increased risk for divorce. The absence of availability during normal social hours defined by our Western society can cause social desynchronization and work-family conflict (118).

The personal and social habits of shift workers can significantly impact their sleep patterns and overall wellbeing. This is true across different age ranges, although specific challenges may vary. Additionally, individual and social determinants of shift work tolerance change with the age of the worker. Younger individuals (18–30) may be more likely to engage in active social lives and late-night social activities, which can sometimes conflict with their shift schedules and the structured recuperation. A marginally elevated risk of excessive drinking among shift workers was often correlated with younger age (119). Younger shift-workers might be more prone to using caffeine and other stimulants to cope with irregular sleep schedules. Moreover, excessive use of screens before bedtime, which is common among younger generations, can contribute to sleep disturbances (120). Due to these displays emitting blue light of around 460 nm spectrum, which suppresses melatonin, release of melatonin is delayed and thus sleep onset as well in the general population as well as in shift workers (121–124). This effect can even last for more than 1 h after discontinuing use (121). When combining display light with general room lighting the side effects worsen. Night mode, meaning reduced brightness, on smartphones might prevent the suppression of melatonin (122). Limited evidence suggests that wearing blue-blocking glasses before bed might aid in diminishing sleep onset latency in workers with variable shift work schedules (125). On the other hand sleep time in general is decreased when using these devices before bedtime through bedtime procrastination (121, 122). Besides, depending on the contents watched, wakefulness might be increased (126). Sleep hygiene with limited and preferably no screen time before bed should thus be advised.

Smartphone apps however can be, thanks to their widespread use, of interest to aid in recovery from shift work through health self-management. Nunes et al. designed a smartphone application, “The Clockwork app” especially made for shift workers that allows them to visualize their sleeping habits, activity level and light exposure at work, but also provides recommendations to promote healthy habits and has a feature to enter their own shift schedule and swap shifts with colleagues (127).

Middle-aged workers (31–50) are more likely to have family responsibilities, such as caring for children or aging parents. Juggling these responsibilities with shift work can lead to disrupted sleep. Older individuals (51+) often experience natural changes in their sleep patterns (128) and shift work can exacerbate these circadian clock changes, making it harder for them to get quality rest (129).

However, social networks are considered an independent determinant of health (114) and there is scientific evidence that social support could play a crucial role in mitigating some of the negative effects of shift work. Having a strong support network, such as friends, family, or colleagues, who understand and empathize with the challenges of shift work can provide emotional support (116, 117, 130, 131). They can offer a listening ear, encouragement and understanding, which can alleviate stress and help individuals cope better. Social support from family to optimize sleeping conditions at home is important as well (132). Additionally, workers who find comfort in religion cope better with the negative effects of shift work as well (130). This support reduces the burden on the individual, allowing them to better manage their responsibilities and reduce stress. Support can also be provided through opportunities for social interaction, reducing feelings of loneliness and enhancing overall wellbeing. Coping with loss of participation can include staying connected by social media and making new friends at work (133). Engaging in activities with friends or participating in social events organized by colleagues can help shift workers maintain a sense of connection and belonging.

Social support can also involve practical assistance, to further ease the combination of family and social life with shift work. Easy access to childcare facilities via the employer or the state, especially when both parents are at work, is vital (118). Family and friends can also step in to help with childcare and household chores. Further practical support via the employer, such as allowing employees to have a say in changes to their shift schedule may also be beneficial. Schedules with fast rotation in contrary to slow rotation may allow regain of social rhythm during parts of the week. There is no hard evidence available pointing to forward or backward rotation as the better choice. Having control over the work schedule improves the ability to attend and participate in social activities and balance the work-family interaction, even when working times are highly irregular and/or no actual change in working hours is observed (118).

Additional social support can be provided in the form of valuable information and advice. Colleagues who have experience with shift work can share strategies for managing sleep, staying healthy, and maintaining work-life balance. This exchange of information can be helpful in adapting to the challenges of shift work and finding effective coping mechanisms (130). Establishing peer support groups specifically for shift workers could be highly beneficial (116, 117). These groups provide a platform for individuals to share their experiences, exchange coping strategies, and offer mutual support. Being part of a community that understands the unique challenges of shift work can significantly improve wellbeing and resilience (130). It's important to note that social support is a two-way street. Individuals must actively seek and foster these connections by reaching out, communicating their needs, and reciprocating support when possible. Building and maintaining a robust social support network can go a long way in mitigating the negative effects of shift work and promoting overall wellbeing.

## 9. Psychotherapy and recovery from shift work

Psychotherapy can be valuable to mitigate some of the negative effects of shift work by addressing the psychological and emotional challenges that can arise from working non-traditional hours. Psychotherapy can provide support and strategies to cope with the challenges associated with shift work and facilitate the recuperation process. It's important to note that psychotherapy should be tailored to an individual's specific needs and circumstances. Consulting with a mental health professional, such as a psychologist or therapist, can help determine the most suitable therapeutic approach to support recovery from the effects of shift work. A therapist can help individuals develop effective coping strategies to manage the unique stressors associated with shift work. This may involve teaching relaxation techniques, stress management skills, and problem-solving techniques. Psychotherapy can also teach stress management techniques, such as mindfulness, cognitive-behavioral therapy (CBT), and problem-solving skills, to help individuals effectively cope with work-related stressors.

As mentioned before, shift work often disrupts the sleep-wake cycle, leading to sleep disturbances and insomnia (131). A higher workload and emotional work can more easily induce these symptoms (134). Psychotherapy can address sleep-related issues by implementing cognitive-behavioral techniques, such as sleep hygiene education, relaxation training, and addressing any underlying anxiety or depression that may contribute to sleep problems. A recent meta-analysis by Reynolds et al. (135) however showed a not significant decrease in mean symptom scores for Insomnia Severity Index (ISI) and Pitsburg Sleep Quality Index (PSQI) after cognitive-behavioral therapy for insomnia (CBTi). This may be explained by the importance of a strict and consistent schedule for behavioral therapy (including sleep restriction and stimulus control therapies) to succeed, which is hard to adhere to when working a rotating schedule. Increased somnolence and reduced vigilance during working hours due to sleep restriction might also pose health risks for workers and diminish adherence even further. Increased compliance can be reached when we take these considerations into account. There is a need for studies that implement tailored CBTi interventions for this specific population. On the one hand study design might benefit from patients' input, on the other hand types of CBTi that work faster such as Intensive Sleep Retraining need to be investigated (135). Sleep restriction therapy could also be implemented in a stepwise manner and patients that suffer from increased somnolence as stated above might benefit from a more flexible sleep window (132).

In a recent study, Li et al. (136) found that nurses that work in shifts are at greater risk for depression and anxiety. A meta-analysis by Lee et al. (137) showed a 40% risk increase for depression for night shift workers regardless of gender, occupation or shift duration. Psychotherapy can provide a safe space for individuals to express their emotions, process work-related challenges, and develop emotional regulation skills. There is very limited evidence showing a positive effect of mental health interventions such as mindfulness and meditation-based interventions available for workers in general (138, 139). Sadly, there is no data available for shift workers in particular.

As shift work has been associated with increased risk of various health issues, such as cardiovascular problems, obesity, and gastrointestinal disorders (16, 140–142) psychotherapy can help promote health and improve work-life balance by guiding individuals to adopt healthier lifestyle habits, such as proper nutrition, regular exercise, and stress reduction. Furthermore, balancing work and personal life can be particularly challenging for shift workers. Shift work requires significant adjustments in one's lifestyle and social interactions. A therapist can assist in navigating these adjustments and provide guidance on maintaining work-life balance, managing relationships and time effectively, setting boundaries to ensure overall wellbeing and developing strategies to create a more balanced and fulfilling lifestyle.

There is very scarce literature about health promotion in shift workers. Data about a consultancy agency providing a 4-h workshop including advice about healthy food options and advice on work-life balance as well as how to improve alertness and sleep quality and reduce sleepiness, showed a higher number of participants reporting an improved feeling of overall health, mainly less gastrointestinal complaints, believing to have found a better work-life balance and having more sleep time, after the course. The data that is available is promising though only related to at work health promotion courses (143). Psychotherapy can offer the patient more than only health promotion as stated in this review and can offer anonymity. Therapeutic interventions, such as cognitive training exercises and techniques to improve focus and mental clarity, can be incorporated into the treatment plan. Scientific data to support this advice in shift workers are to our knowledge not available and are therefore needed in the future. Psychotherapy can help in managing and treating shift-work disorder through a combination of behavioral interventions, sleep hygiene strategies, and possibly referral to a sleep specialist if necessary. Online and in-patient formats can be equally successful. An online CBT-I treatment course would benefit workers who would prefer to stay anonymous and have difficulties attending therapy sessions at certain daytime hours (144).

It's important to note that the specific approach and techniques used in psychotherapy will depend on the individual's needs and the therapist's expertise. Consulting with a qualified mental health professional will ensure personalized and effective support for managing the challenges associated with shift work.

## 10. Conclusion

Shift work often involves irregular working hours, disrupting the body's natural circadian rhythm and placing significant strain on individuals. As a result, there is a pressing need for recuperation after engaging in such work. The human body thrives on stability and routine, and when this balance is disrupted, it can lead to a myriad of physical and mental health issues. Post-shift recuperation is essential to allow workers to restore their energy levels, promote restorative sleep, and maintain overall wellbeing. It provides a valuable opportunity for individuals to engage in activities that promote relaxation, self-care, and stress reduction, helping them recover from the physical and psychological demands of shift work. Recuperation periods also allow workers to spend quality time with family and friends, fostering social connections and enhancing their overall quality of life. Furthermore, adequate rest and recovery enable individuals to return to work feeling refreshed, rejuvenated, and better equipped to perform at their best, ultimately improving productivity and job satisfaction. In conclusion, the need for recuperation after shift work is undeniable, as it plays a crucial role in maintaining the health, wellbeing, and overall effectiveness of shift workers.

## Author contributions

SD: Data curation, Writing—original draft, Writing—review & editing. AK: Conceptualization, Data curation, Methodology, Supervision, Writing—original draft, Writing—review & editing. DP: Data curation, Writing—original draft, Writing—review & editing. DT: Supervision, Writing—review & editing. BB: Supervision, Writing—review & editing.
